# Recruitment of gastroenterology trainees with the help of a new training model?

**DOI:** 10.1055/a-2081-9458

**Published:** 2023-05-15

**Authors:** Hiroki Kato, Makoto Kobayashi, Motoyoshi Yano

**Affiliations:** Department of Gastroenterology, Yokkaichi Municipal Hospital, Mie, Japan

Recruitment of trainees is necessary to provide the number of gastroenterologists needed. Compared to other branches of internal medicine, gastroenterology involves many procedures, and it is thought that a sense of joy in performing procedures is a main reason why young doctors become gastroenterologists.

A training model for upper gastrointestinal endoscopy is often used for the instruction of trainees 1 . The recently developed EASY ( *E* ndoscopist and *A* ssistant’s *S* imulator dr *Y* lab) (Tanac Co., Ltd., Gifu, Japan) is an endoscopic simulator developed by Matsuzaki and Tsunemi that is capable of undergoing resection with a snare and clip closure ( Fig. 1 , Fig. 2 ). We instructed trainees in endoscopic procedures using an upper gastrointestinal endoscopy model and the EASY and investigated whether the latter would be a useful instrument for encouraging trainees to join our Department of Gastroenterology ( Video 1 ).

**Fig. 1:**
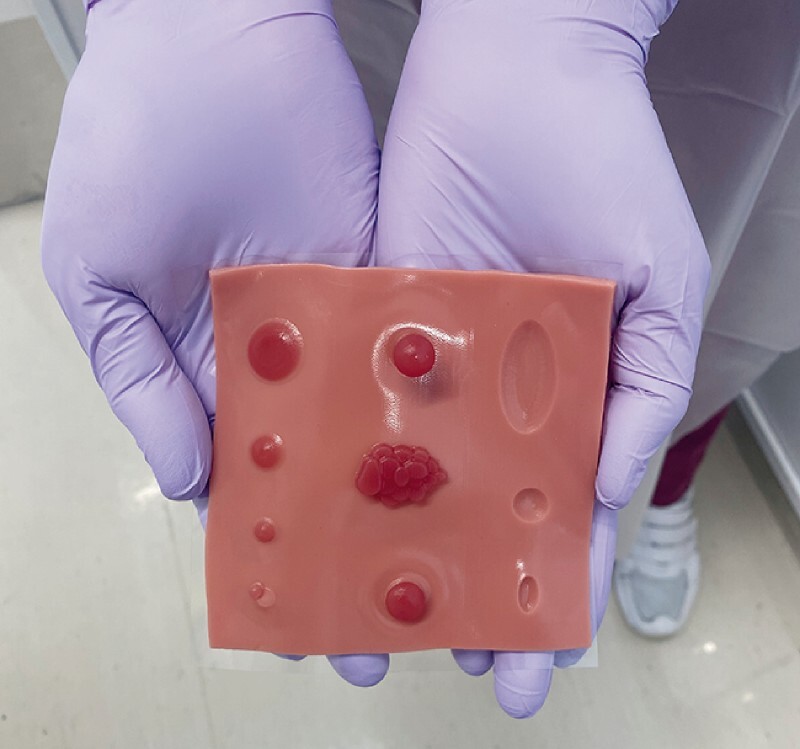
The recently developed EASY ( *E* ndoscopist and *A* ssistant’s *S* imulator dr *Y* lab): a new endoscopy training model.

**Fig. 2:**
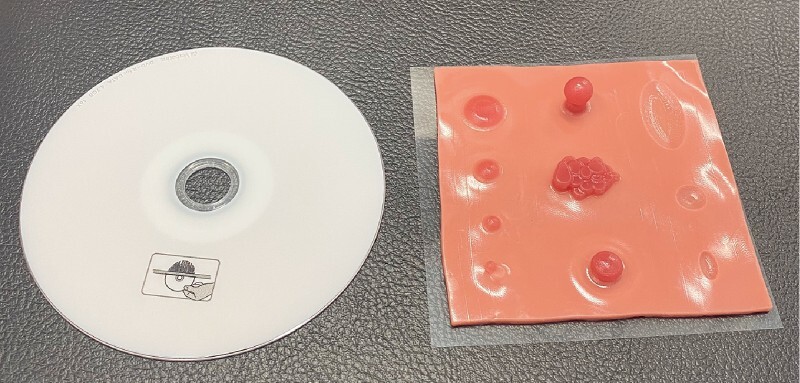
The new training model is about the same size as a compact disc.

**Video 1** Trial run for recruiting trainees to gastroenterology with the help of a new endoscopy training model.


Using the upper gastrointestinal endoscopy training model and the EASY, we explained the procedure and provided actual training to 10 junior residents in our hospital. A questionnaire was administered after the training, in which participants’ level of understanding and satisfaction in relation to the endoscopic procedures were evaluated on a 5-point scale.

For the upper gastrointestinal endoscopy model, the comprehension level was 3.80 ± 0.40 and the satisfaction level was 3.50 ± 0.72. For the EASY, the comprehension level was 4.10 ± 0.32 and the satisfaction level 4.60 ± 0.27. Thus, the satisfaction level was higher with EASY than with the upper gastrointestinal endoscopy model ( *P* < 0.05); there were no significant differences in comprehension level ( *P* > 0.05).

The EASY is a dry lab that does not use living tissue, and the reason for the high satisfaction rating was that even junior residents could perform polypectomy and clipping “realistically” and “enjoyably.” Residents showed a high level of satisfaction with the EASY.

We believe that using the EASY endoscopy simulator in addition to the endoscopy training model may be useful for training and recruitment of endoscopists.

Endoscopy_UCTN_Code_TTT_1AU_2AB

## Notes

Endoscopy E-Videos

https://eref.thieme.de/e-videos
*E-Videos* is an open access online section of the journal *Endoscopy* , reporting on interesting cases and new techniques in gastroenterological endoscopy. All papers include a high-quality video and are published with a Creative Commons CC-BY license. Endoscopy E-Videos qualify for HINARI discounts and waivers and eligibility is automatically checked during the submission process. We grant 100% waivers to articles whose corresponding authors are based in Group A countries and 50% waivers to those who are based in Group B countries as classified by Research4Life (see: https://www.research4life.org/access/eligibility/ ). This section has its own submission website at https://mc.manuscriptcentral.com/e-videos
